# Transcriptome and metabolome analyses revealing the potential mechanism of seed germination in *Polygonatum cyrtonema*

**DOI:** 10.1038/s41598-021-91598-1

**Published:** 2021-06-09

**Authors:** Rong Liu, Jing Lu, Jiayi Xing, Mei Du, Mingxiu Wang, Lei Zhang, Yunfang Li, Chihong Zhang, Yu Wu

**Affiliations:** 1 Chengdu Institute of Biology, Chinese Academy of Sciences, Chengdu, 610041 China; 2 College of Life Sciences, Sichuan University, Chengdu, 610065 China; 3 University of Chinese Academy of Sciences, Beijing, 100049 China; 4 Innovation Academy for Seed Design, CAS, Beijing, 100101 China

**Keywords:** RNA metabolism, Transcriptomics, Biochemistry, Developmental biology, Plant sciences

## Abstract

*Polygonatum cyrtonema* Hua (Huangjing, HJ) has medicinal and edible value in China. However, the seeds of this plant are naturally difficult to germinate. Therefore, to elucidate the mechanism underlying the germination of this plant in order to meet the market demand, the metabolomic and transcriptomic analyses were performed in this study. We observed that plant hormones and α-amylase activity were differentially regulated when comparing germinated and un-germinated seeds. In addition, the metabolites related to phenylpropanoid and flavonoid biosynthesis were significantly up-accumulated in germinated seeds. Hydroxycinnamoyl derivatives and organic acids were observed to be significantly decreased during germination. The results of this study suggested that compared to un-germinated seeds, germinated seeds promote flavonoid synthesis and inhibit lignin synthesis which could be beneficial to the germination of HJ seeds. Furthermore, these results suggested that starch if hydrolyzed into glucose, which could provide the necessary energy for germination. Our results may help to establish a foundation for further research investigating the regulatory networks of seed germination and may facilitate the propagation of HJ seeds.

## Introduction

*Polygonatum cyrtonema* Hua (Huangjing in Chinese) is a species in the family Liliaceae, that is widely grown in China. Due to the unique medicinal and edible value of this plant, Huangjing (HJ) has been used for more than 2000 years. HJ has also been utilized as a treatment for weakness, and its properties include anti-inflammatory, antimicrobial, hypoglycemic, and immunoregulatory effects^1^.

Previous studies have focused on the resource investigation and cultivation^2^, rhizome tissue culture^3^, chemical composition^4^ and pharmacological value^5^ of HJ. However, little information regarding seed germination is available. HJ seeds are naturally difficult to germinate. Under conventional sowing conditions, HJ seeds require more than 6 months of dormancy to germinate^6^. Considering the rapid development of the HJ cultivation industry in China. Therefore, its reproductive and growth cycles should be accelerated through artificial technologies.

Seed germination is tightly regulated by transcriptional and metabolic changes. Increasing information from transcriptomics and metabolomics studies has enhanced global understanding of the germination process and regulation. Metabolomics has recently become a major research tool to analyze substrates and products in metabolic pathways^7,8^. Many studies have also attempted to develop the variations in gene expression and build comprehensive models of seed germination^9^. A number of studies also regarding metabolism during seed germination have also been conducted in Arabidopsis^10^, barley^11^ and rice^12,13^. In *Polygonatum cyrtonema*, to the best of our knowledge, the metabolomic and transcriptomic profiling of seed germination have not been reported to date.

Germination is regulated by multiple endogenous factors, such as plant hormones, and by environmental conditions^14,15^. The phytohormones gibberellin acid (GA) and abscisic acid (ABA) play crucial roles in the regulation of seed germination by stimulating or suppressing seed germination^16^. In *Polygonatum cyrtonema*, carbohydrates and proteins stored in the endosperm are mobilized during seed germination to provide energy and substrates for developing seedlings^17^. During seed germination, bioactive GAs are synthesized and transported to induce α-amylase gene expression and α-amylase synthesis^18,19^. Next, α-amylase is secreted into the endosperm to hydrolyze the stored starch^20^. At the same time, organic acids and flavonoids may participate in acidifying the endosperm tissue, releasing stored starch into the metabolism to affect seed germination^21,22^.

However, metabolomics and transcriptomic profiling during HJ seed germination have not been performed to date. To elucidate the mechanism underlying HJ seed germination, we performed the first integrated metabolomics and transcriptomics analysis of the mechanism governing HJ seed germination using liquid chromatography–MS (LC/MS) and next generation sequencing (NGS). The gene expression and metabolites associated with starch and sucrose metabolism, flavonoid biosynthesis, plant hormone transduction, phenylpropanoid metabolism and α-amylase activity were investigated in this study. Our results may help to elucidate the biochemical mechanisms underlying *Polygonatum cyrtonema* seed germination at the metabolomic and transcriptomic levels.

## Results

### Morphological identification and phenotype of *Polygonatum cyrtonema*

**Figure 1:**
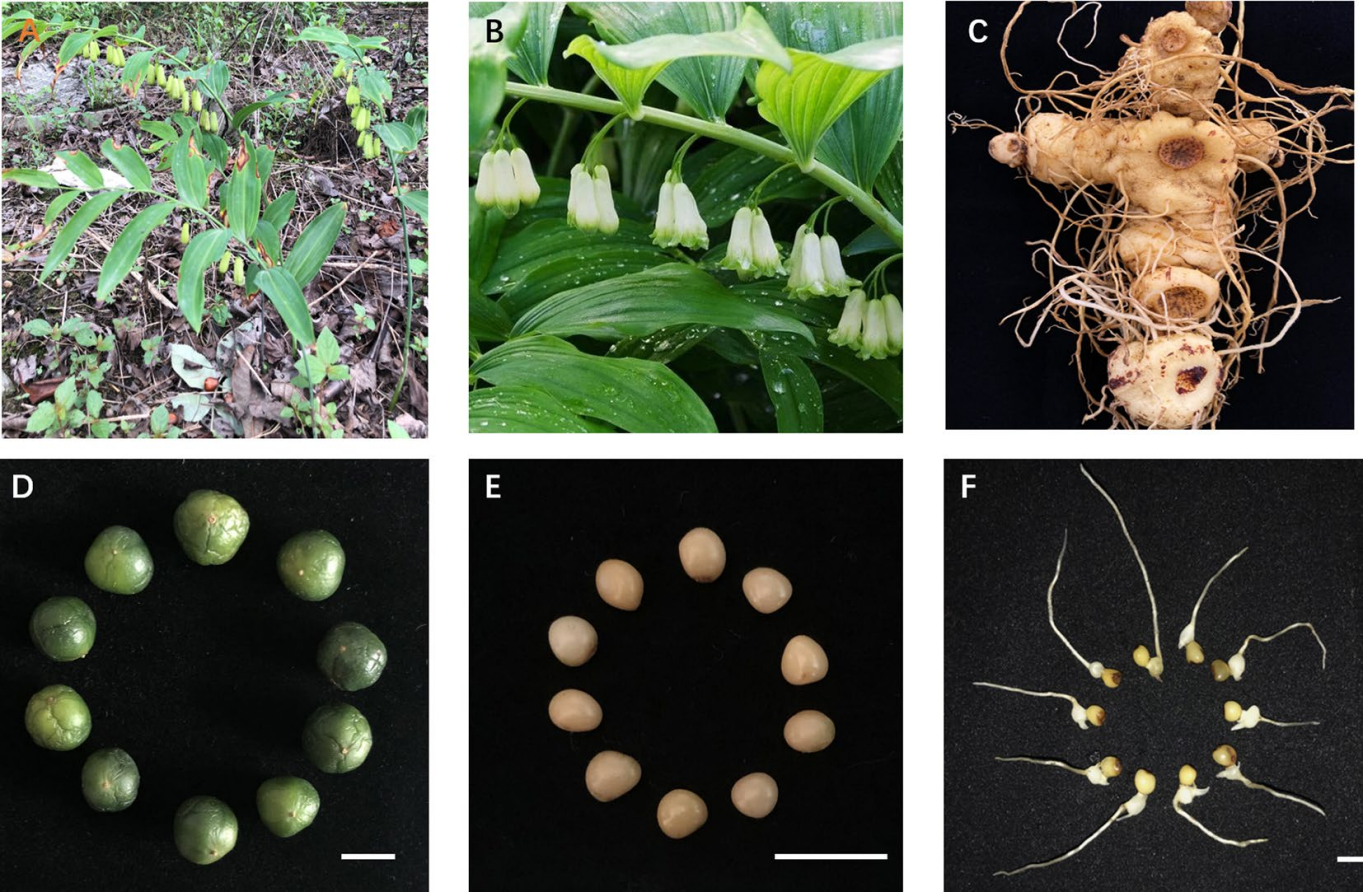
The plant morphology (**A**), flower (**B**), tubers (**C**), fruits (**D**), seeds (**E**) and germinated seeds (**F**) of *Polygonatum cyrtonema* Hua. The bar = 1 cm.

The plant morphology, flowers and tubers of HJ are shown in Fig. 1A,B and C. Figure 1B and C were obtained from the Plant Photo Bank of China, PPBC (http://ppbc.iplant.cn/). *Polygonatum cyrtonema* Hua is a perennial herb of the Liliaceae family, with thick rhizomes, alternate phyllotaxy and yellow-green perianth, this plant flowers from May to June and fruiting from August to October. The medicinal part of *Polygonatum* is the rhizome. In this study, the fruits and seeds (Fig. 1D and E) of *Polygonatum cyrtonema* Hua were obtained from Chengdu Institute of Biology, Chinese Academy of Sciences. Germinated seeds (radicle and embryo elongation stage) were collected for transcriptome sequencing and metabolome analysis after six months of planting (Fig. 1F).

### RNA-seq de novo assembly and function annotation of *Polygonatum cyrtonema*

Six cDNA libraries (CK(control check GA)1, CK2, CK3, G1, G2, and G3) were sequenced by Illumina HiSeq to detect gene expression information during seed germination. A summary of RNA-Seq data is shown in Table 1. High-quality and clean reads were obtained by filtering low-quality reads. The Q30 of six samples exceeded 93% and the GC was 50.36%. A total of 54,531 unigenes were obtained after de novo assembly using Trinity software. The number of unigenes annotated in NCBI non-redundant(NR) were 28,678 (52.59%), 26,808 (49.16%) were obtained from eggNOG database, 21,572 (39.56%) annotated in SwissProt, 11,521 (21.13%) were annotated in KEGG, 21,252 (38.97%) were annotated in gene ontology (GO) and 18,292 (33.55%) were annotated in eukaryotic complete genomes (KOG). A total of 31,724 unigenes were over 500 bp and 14,323 unigenes were over 1000 bp. The average length of unigenes was 875 bp, and the length of N50 was 1,138 bp. The assembly quality of the transcriptome was determined to be conducive to reliable RNA-seq results.

**Table 1:** Statistics and functional annotations of unigenes in 6 RNA sequencing libraries.

	Number of unigenes	Percentage (%)
> 300 bp	54,531	100
≥ 500 bp	31,724	58.2
≥ 1000 bp	14,232	26.1
N50	1138 (bp)	–
Max length	12,315 (bp)	–
Min length	322 (bp)	–
Average length	875 (bp)	–
NR	28,678	52.6
EggNOG	26,808	49.2
SwissProt	21,575	39.6
KEGG	11,521	21.1
GO	21,252	38.9
KOG	18,292	33.6
Total	54,531	100

### Comparative analysis and qRT-PCR verification

To elucidate the molecular basis for HJ seed germination, comparative transcriptomic analysis was conducted. Differential expression unigenes (DEGs) were analyzed using the FPKM method to determine the degree of between the two seed groups. Compared with the CK, a total of 18,308 up-regulated DEGs and 9,709 down-regulated DEGs were found in the germinated seeds (Fig. S1A, Supplemental File 1). The results imply that these 27,017 DEGs might play roles in germination.

To evaluate the accuracy of transcriptome profiles from the RNA sequencing analysis, 18 genes related to seed germination were selected for qRT-PCR expression analysis. The correlation of RNA-Seq (FPKM) and qRT-PCR are shown in Figure S1B. The relative expression levels of the genes from qRT-PCR were consistent with those from the RNA-Seq data. There was a significantly positive correlation between the RNA-seq and qRT-PCR results (p < 0.01), which indicated that the RNA-seq data were reliable.

### Kyoto encyclopedia of genes and genomes (KEGG) analysis of DEGs

**Figure 2:**
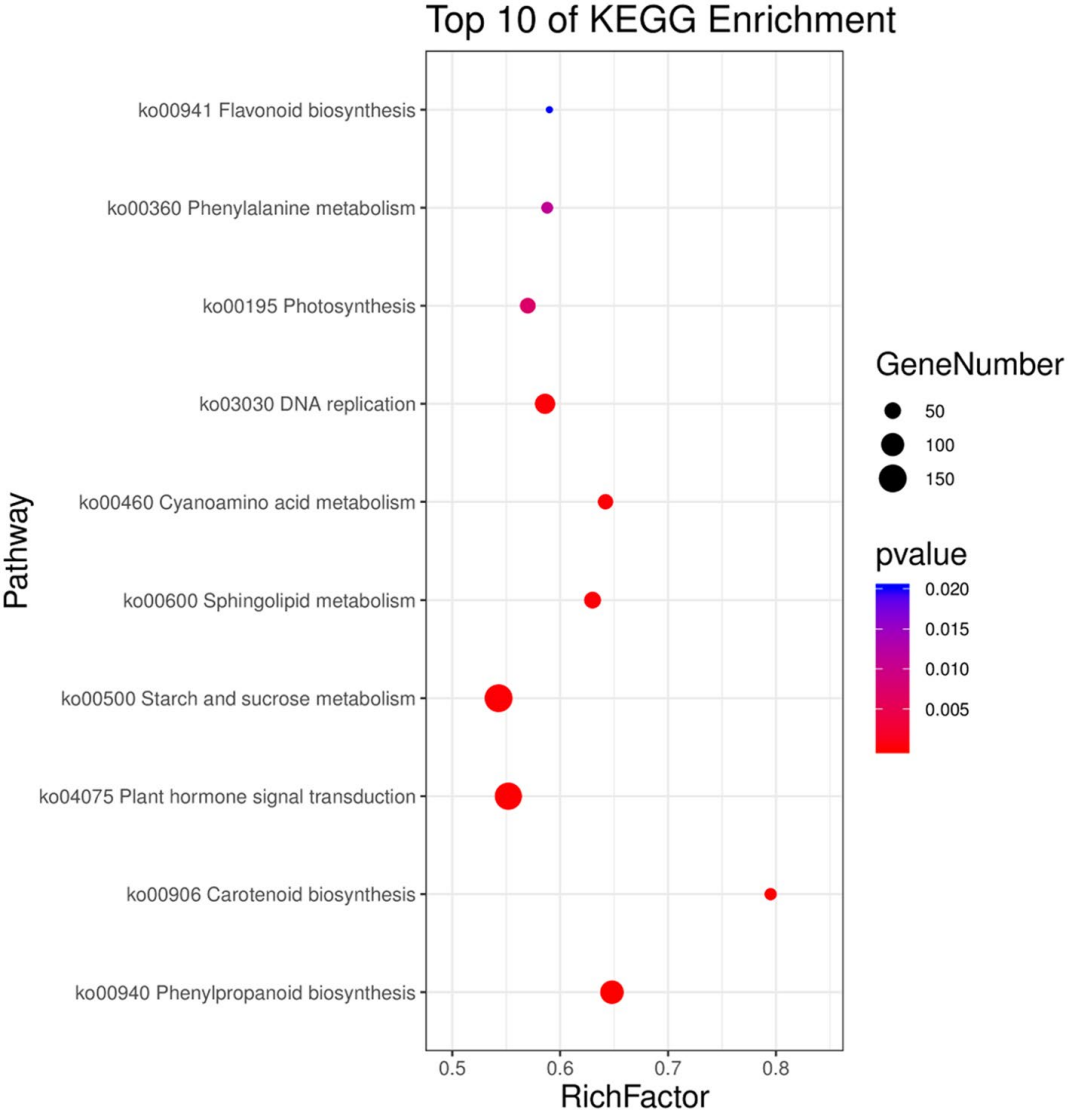
The top ten of KEGG enrichment of differentially expressed unigenes (DEGs) in *Polygonatum cyrtonema* Hua seeds.

To identify the major signaling pathways represented by DEGs, enrichment of DEGs in the KEGG pathway^23–25^ was analyzed at a significance level of *p* < 0.05. The KEGG annotations indicated that plant hormone signal transduction (ko04075), phenylpropanoid biosynthesis (ko00940), starch and sucrose metabolism (ko00500), phenylalanine metabolism (ko00360), flavonoid biosynthesis (Ko00941) pathways were significantly enriched between the G and CK groups (Fig. 2, Supplemental File 2).

### DEGs of phenylpropanoid and flavonoid biosynthesis related to seed germination

**Figure 3:**
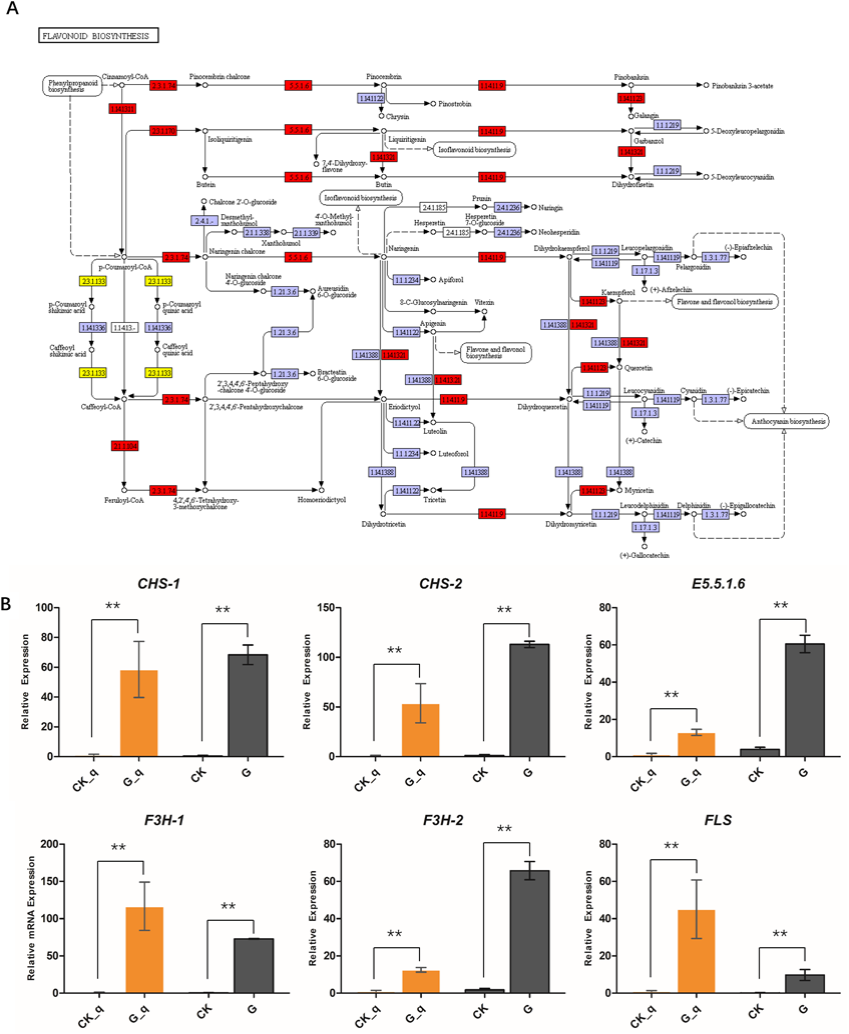
The expression of DEGs enrichment in flavonoid biosynthesis (Ko00941, cited from Kanehisa laboratories) in G vs CK seeds (**A**), genes marked with red indicate up-regulated, yellow has both up- and down-regulated genes in germinated seeds. The expression of flavonoid biosynthesis pathway genes by qPCR determination (**B**), expression levels estimated using log2(fold change) for each transcript (**p < 0.01). In the histogram, CK_q and G_q represents qPCR analysis; CK and G represent the FPKM value of RNA_seq.

The DEGs involved in phenylpropanoid biosynthesis (ko00940) and flavonoid biosynthesis (Ko00941) were examined in this study to determine their involvement in seed germination. A total of 114 DEGs (91 up-regulated, 23 down-regulated) were detected in phenylpropanoid biosynthesis, including phenylalaninammo-nialyase (PAL), trans-cinnamate 4-monooxygenase (CYP73A), 4-coumarate-CoA ligase (4CL) and cinnamoyl-CoA reductase (CCR), etc. 22 DEGs were enriched in flavonoid biosynthesis and mostly up-regulated, such as chalcone synthase (CHS), flavonol synthase (FLS), flavanone 3-hydroxylase (F3H) and chalcone isomerase (CHI) et al. (Fig. 3A, Supplemental File 1). To validate the transcriptome expression data for genes involved in flavonoid biosynthesis, we randomly selected six genes for qPCR analysis. These genes included CHS (TRINITY_DN14701_c0_g2_i1_4, TRINITY_DN27324_c0_g1_i1_3), E5.5.1.6/Chalcone isomerase (TRINITY_DN39749_c0_g1_i5_3), F3H (TRINITY_DN16505_c0_g2_i1_4, TRINITY_DN16337_c0_g1_i1_4), and FLS (TRINITY_DN43663_c0_g1_i1_3). The fold changes of RNA_seq were all consistent with those obtained from the qPCR analysis (Fig. 3B). These results indicate that the phenylpropanoid and flavonoid biosynthesis pathways are important for seed germination.

### DEGs related to starch and sucrose metabolism in seed germination

**Figure 4:**
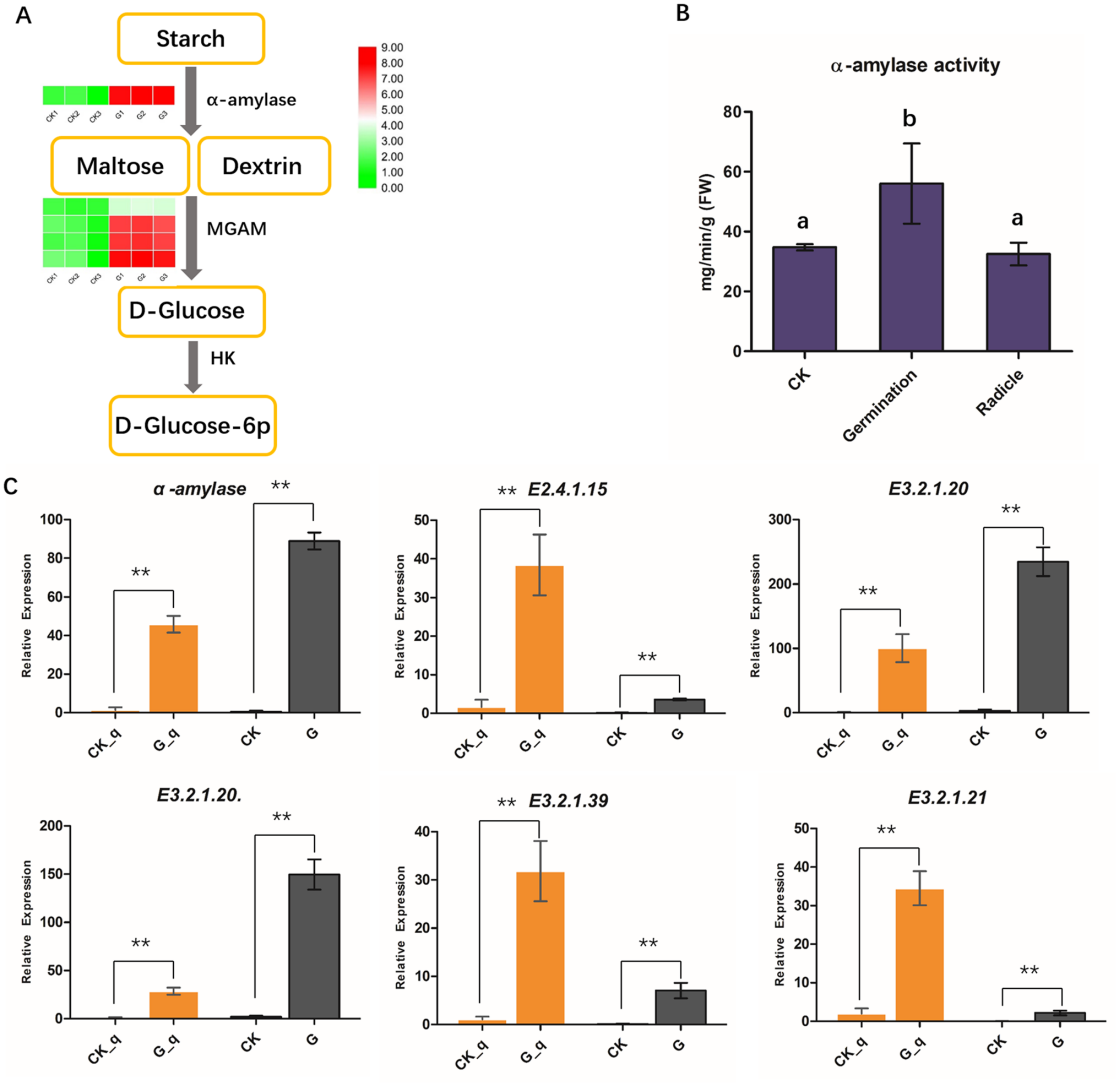
The expression of starch and sucrose metabolism pathway genes in *Polygonatum cyrtonema* Hua (**A**), expression levels are indicated by the heatmap at G and CK group, estimated using log2(fold change) for each transcript. The α-amylase activity (**B**) and the enzyme genes determined by qRT-PCR (**C**). In the histogram, CK_q and G_q represents qPCR analysis; CK and G represent the FPKM value of RNA_seq (**p < 0.01).

A significantly enriched pathway was found in G vs CK seeds, and a total of 59 DEGs were detected in starch and sucrose metabolism (ko00500), specifically 41 up- and 18 down-regulated (Supplemental File 3) in HJ. We found that the α-amylase synthesize gene and maltase-glucoamylase (MGAM) enzyme genes were significantly up-regulated during seed germination (Fig. 4A). Therefore, we determined the α-amylase activity and related gene expression in this pathway. The results showed that the highest α-amylase activity was found in germinated seeds, followed by the CK seeds and radicles (Fig. 4B).The α-amylase synthesize gene, E2.4.1.15 (trehalose 6-phosphate synthase), E3.2.1.20 (MGAM), E3.2.1.39 (glucan endo-1,3-beta-D-glucosidase, EGLC) and E3.2.1.21 (beta-glucosidase) in starch and sucrose metabolism were characterized by qRT-PCR (Fig. 4C) and all DEGs were significantly up-regulated in germinated seeds. The expression levels of unigenes were consistent with the trends of α-amylase activity, suggesting that the activity of α-amylase and sucrose metabolism might play important roles in the completion of germination and seedling establishment.

### Plant hormone related DEGs in seed germination

**Figure 5:**
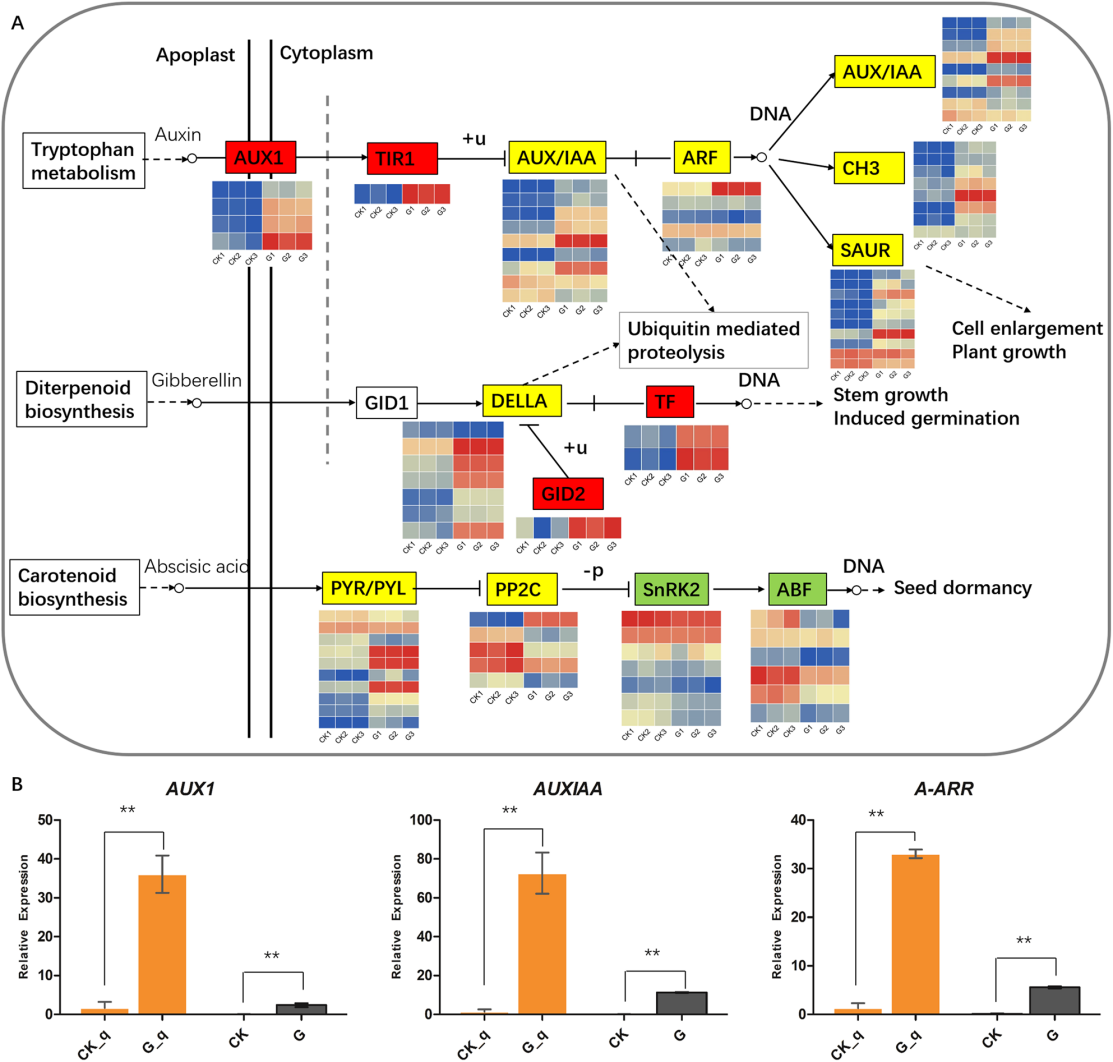
The DEGs expression enriched in auxin, gibberellin (GA) and abscisic acid (ABA) signaling pathways (**A**), expression levels are indicated by the heatmap at G and CK group, estimated using log2(fold change) for each transcript. The genes in the box stained with red indicate up-regulated, the box stained with green was down-regulated, the box stained with yellow has both up- and down-regulated genes in germinated seeds. Certain key genes in those pathways were determined by qRT-PCR (**B**). In the histogram, CK_q and G_q represents qPCR analysis; CK and G represent the FPKM value of RNA_seq (**p < 0.01).

In this study, we found that plant hormone signal transduction (ko04075) was greatly enriched, indicating that plant hormones play an important role in regulating the germination of plant seeds. DEGs involved in the auxin synthesis regulation pathway, such as auxin influx carrier (AUX1 LAX family) (AUX1) and transport inhibitor response (TIR1) were significantly up-regulated in germinated seeds (Fig. 5A, Supplemental File 4). The F-box protein GID2 (GID2) and transcription factors (TFs) (PIF3 and PIF4) in the GA related pathway were also up-regulated. However, when compared to CK, DEGs in the ABA pathway were almost all down-regulated in G including protein phosphatase 2C (PP2C), serine/threonine-protein kinase SRK2 (SnRK2) and ABA responsive element binding factor (ABF). In addition, we found that the DEGs were significantly changed in the upstream of salicylic acid synthesis (ko00360, ko00362), while some genes in the downstream of salicylic acid pathway were also significantly different, such as regulatory protein NPR1(NPR1) and pathogenesis-related protein 1(PR1). To validate the transcriptome expression data, three plant hormone signal transduction genes, AUX1 (TRINITY_DN27428_c0_g1_i1_3), auxin-responsive protein IAA (AUX/IAA) (TRINITY_DN32072_c0_g1_i1_2) and two-component response regulator ARR-A family (A-ARR) (TRINITY_DN21518_c0_g1_i1_2) were randomly selected for qPCR analysis (Fig. 5B). The fold changes for the qPCR analysis indicated the relative expression of the germinated and the control seeds, and the fold changes of RNA_seq were consistent with the qPCR analysis. Therefore, we suggest that auxin, GA and ABA are involved in seed germination.

### Metabolomic analysis and association with transcriptomic analysis

To verify the accuracy of the transcriptomic sequencing results, we conducted metabolomic analysis. In this study, a total of 637 metabolites were detected in HJ seeds by LC–MS, including certain primary and secondary metabolites, such as organic acids, amino acids, flavonoids, and lipids. Compared with the control, 230 different metabolites (96 up-regulated and 134 down-regulated) were detected in germinated seeds. (Figure S2). To study the association between transcriptome and metabolome in seeds, we analyzed the connection of DEG expression and metabolites. Certain key enzymes encoding DEGs were related to metabolite contents, indicating that these enzymes encoded by DEGs might be involved in seed germination.

### Flavonoids and hydroxycinnamyl related to seed germination

**Figure 6:**
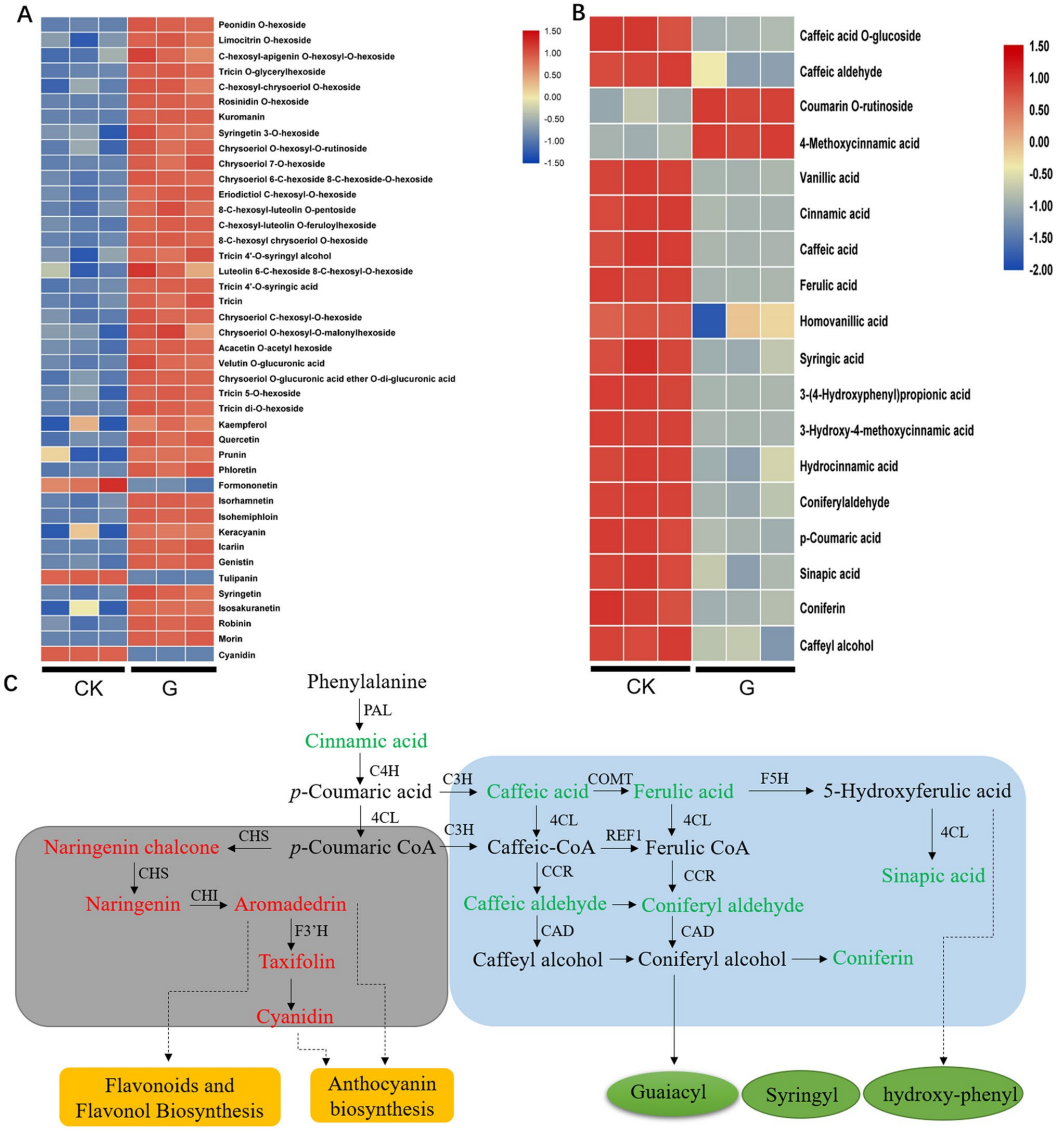
The differentially metabolites of flavonoids (**A**) and Hydroxycinnamoyl derivatives (**B**) between germinated and control seeds in *Polygonatum cyrtonema* Hua. Accumulation of differential metabolites in flavonoid biosynthesis and phenylpropanoid metabolism pathway in germinated vs CK seeds (**C**). The metabolites in the (**C**) stained with red indicate up-accumulated, stained with green has down-regulated in germinated seeds.

We conducted a detailed analysis of the different metabolites in G vs CK seeds. The results showed that 42 flavonoids significantly accumulated during the germination of seeds (Fig. 6A, Supplemental File 5-1). Compared with CK, all 42 flavonoids were significantly increased in the G group, including flavanone, flavonol and anthocyanins, etc. The result is consistent with the RNA_seq results. Therefore, we suggesting that the synthesis of flavonoids may play an important role in the germination of HJ seeds.

In addition, we found that hydroxycinnamoyl derivatives showed a significant down-regulation trend (Fig. 6B, Supplemental File 5-2). Hydroxycinnamoyl derivatives are precursors in the lignin synthesis, and we speculated that the metabolites in this pathway might be negatively regulate the germination of seeds. Therefore, we further analyzed the flavonoid synthesis and the lignin synthesis pathway and found that both pathways are derived from the phenylalanine metabolism pathway. Based on the KEGG pathway, we mapped metabolic pathways related to germination of HJs. The metabolites of the flavonoid synthesis pathway, specifically naringenin chalcone, naringenin, aromadendrin, taxifolin and cyanidin were significantly increased in germinated seeds. However, compared with CK, the metabolites cinnamic acid, caffeic acid, caffeic aldehyde, coniferyl aldehyde, coniferin and sinapic acid were significantly decreased in the phenylpropanoid pathway (Fig. 6C). The results showed that the metabolite flow of the phenylalanine pathway was mainly to enhance flavonoid synthesis and to reduce the lignin synthesis during the seed germination, which might have been beneficial to the germination of HJ seeds.

### Organic acids related to germination of *Polygonatum cyrtonema*

In the present study, we found that organic acids were significantly down-regulated with the germination of HJ seeds (Fig. S3, Supplemental Files 5-3). The results suggested that organic acids could significantly inhibit HJ seed germination. We performed further analysis and found that benzoic acid, ferulic acid, and coumaric acid were the most significant organic acid metabolites during germination. Therefore, we speculated that these metabolites might have a major inhibitory effect on the germination of HJ seeds.

## Discussion

Seeds are at the core stage of agriculture and biodiversity for plant species survival. Seed germination, an adaptive trait, is a crucial phase in the plant life cycle which is strongly related to the seedling survival rate and subsequent growth. Germination commences with water uptake by the quiescent dry seed and ends with visible radicle elongation through the seed coat^26^. According to Baskin et al.^27^ in the criteria for the classification of seed dormancy, HJs belong to morphological and physiological dormancy.

Breaking the dormancy of seeds and shortening the breeding cycle, which can help to meet the demand for seedling supply of HJ. In this study, transcriptomic analysis combined with metabolomic analysis was used to detect the changes in RNA level and metabolome levels in the seed germination of HJs. We found that the DEGs were significantly enriched in the flavonoid synthesis and the phenylpropanoid metabolism pathway. DEGs were significantly up-regulated in the flavonoid (anthocyanin) synthesis pathway. The results of metabolites were also consistent with the RNA_seq profile. Compared with the control, the accumulation of flavonoids significantly increased, while the hydroxycinnamoyl derivatives significantly decreased in germinated seeds. Flavones are synthesized by the flavonoid pathway, which is part of phenylpropanoid metabolism^28^. Certain studies have provided evidence to suggest that flavonoids play an important role in plant defense against biotic and abiotic stresses, such as the plant defense response to pathogens^29^, oxidative damage^30^ and UV stress^31^ etc. However, few studies have analyzed the overall transcriptomic and metabolomic changes of the flavonoid biosynthesis pathway in seed germination of HJs. Our results suggest that the DEGs and the metabolites of flavonoid synthesis pathway can promote seed germination of HJ.

The seed coat of HJ seedd is constituted by a layer of lignified cells^32^, which prevents water absorption and has an inhibitory effect on seed germination. Lignin, a plant secondary metabolite that is a product of the phenylpropanoid pathway, represents a physical barrier to seed germination. In the present study, we found that DEGs and metabolism were significantly down-regulated in the phenylpropanoid synthesis (lignin synthesis) pathway. Phenylpropanoid biosynthesis pathway begins with phenylalanine and proceeds with various steps of the pathway catalyzed by seven types of enzymes: PAL, cinnamate 4-hydroxylase (C4H), 4CL, CCR, caffeoyl-CoA Omethyltransferase (CoMT), ferulic acid 5-hydroxylase (F5H) and cinnamyl alcohol dehydrogenase (CAD). Compared with CK, the metabolites were down-regulated significantly in this pathway (Fig. 6C). Finally, the products of three types of monolignols polymerize to form lignin, such as guaiacyl (G), syringyl (S), and hydroxyphenyl (H) lignin, which decreased in germinated seeds. Therefore, the reduction of metabolites in the phenylpropanoid synthesis pathway leads to a decrease in lignin synthesis, which is also conducive to the HJ seeds to break through the seed coat and breaking dormancy.

Germination is regulated by plant hormones^33^. HJ seeds might also be affected by endogenous hormones and physiological after-ripening, which leads seeds to exhibit a low germination rate and difficult emergence of seeds. Previous study reported that plant hormones, after-ripening and gibberellin (GA) are known to be involved in seed germination in other species^34^. GA homeostasis plays important roles in regulating seed germination, plant growth and development^35^. GA induces cell wall-decomposing enzymes to accelerate endosperm degradation, which is a prerequisite for seed germination^36^. GA homeostasis is controlled by GA metabolism, including biosynthesis and inactivation^37^. Abscisic acid (ABA) also plays an important role in regulating seed germination. GA and abscisic acid (ABA) are recognized as the major hormones that have antagonistic effects on the regulation of seed germination^38^. ABA is involved in the induction and the maintenance of seed dormancy, while GA and auxin are positive regulators of seed germination^39^. Similar to previous study, our results also found that GA- and auxin-related DEGs were significantly more highly expressed in germinated seeds. However, the expression of DEGs associated with ABA signal transduction was significantly down-regulated. Both GA and auxin can promote germination to break seed dormancy, while ABA promotes seed dormancy. Therefore, we speculated that these DEGs play an important role in regulating seed dormancy and germination.

In HJ seeds, carbohydrates and proteins are stored in the endosperm to provide energy and substrates for germination. Seed germination is dependent on the degradation of storage reserves in mature seeds, and the sugars from starch hydrolysis are the major source of energy for seedling emergence^40^. HJ seeds have a high density of endosperm and a thick endosperm coat, which contain a large number of starch (> 73.7%)^41^. Seed germination is related to starch hydrolysis in endosperm. In the process of seed germination, the decomposition of starch can promote the seed germination^42^. The α-amylase (EC 3.2.1.1) is the major enzyme involved in the hydrolysis of starch to glucose^43^. Thus, α-amylase activity is an important factor in seed germination^44^. During seed germination, bioactive GAs is synthesized in the embryo and transported to the aleurone layer to induce α-amylase gene expression and α-amylase synthesis. Then, α-amylase is secreted into the endosperm to hydrolyze the stored starch. The activity of α-amylase is one of the indicators for judging seed germination. Studies found that positive relationships between α-amylase activity and the rice seed germination rate. Our results demonstrate that α-amylase activity significantly increased in germinated seeds and that the DEGs were significantly upregulated in the starch sugar metabolism pathway. This result suggests that hydrolysis of starch to monosaccharides also provides necessary energy for seed germination also in HJ.

## Conclusions

In this study, we performed the metabolomics and transcriptomics analyses to reveal the underlying mechanisms governing *Polygonatum cyrtonema* Hua involved in maintaining seed dormancy. A total of 54,531 unigenes were identified by RNA_seq and the average length of the unigenes was 875 bp, the length of N50 was 1,138 bp. There was a total of 18,308 up-regulated DEGs and 9,709 down-regulated DEGs in the germinated seeds compared to the un-germinated seeds. The metabolites were also detected in HJ seeds by LC–MS. The results of RNA_seq and metabolomic indicate that the phenylpropanoid and flavonoid biosynthesis pathways are important for seed germination. We speculated that the metabolite flow of the phenylalanine pathway was mainly to enhance the flavonoid synthesis and to decease the lignin synthesis during the seed germination, which was beneficial to the germination of seeds. In addition, the results show that plant hormones (auxin, GA and ABA) and organic acids were involved in seed germination. In present study, we found that ABA and organic acids might also be related to seed germination. Moreover, the expression levels of unigenes were also consistent with the trends of α-amylase activity, suggesting that the activity of α-amylase and sucrose metabolism might play an important role during seed germination. To study the association between transcriptomic and metabolic in seeds, we analyzed the connection of DEGs expression and metabolites. Certain key enzyme encoding DEGs were related to metabolite production, this result suggests that these enzymes encoding DEGs and metabolites might be involved in seed germination.

## Materials and methods

### Plant materials

The HJ seeds were used in this study, which undertook the formal identification by professor Yu Wu’s lab at the Chengdu Institute of Biology, Chinese Academy of Sciences (CIB, CAS), and permission was obtained the permission from CIB, CAS. Mature HJ seeds were surface sterilized with 3% H_2_O_2_ for 10 min, then vigorously rinsed with distilled water (> 200 ml/per time) for 5 times and air-dried (floating seeds were removed). Plump and shiny seeds were selected for cultivation in rotten leaf soil for experiments. Germinated and un-germinated (CK) HJ seeds were collected after six-months (each group had 3 biological replicates). Germinated HJ seeds were collected at the germination stage (radicle and embryo elongation). All samples were frozen in liquid nitrogen and stored in a − 80 °C refrigerator for metabolomic analyze and transcriptome sequencing.

### Metabolomic analysis of HJ seeds

The freeze-dried seed was crushed using a mixer mill (MM 400, Retsch) with a zirconia bead for 1.5 min at 30 Hz. One hundred milligrams of powder were weighted and extracted overnight at 4℃ with 1.0 ml 70% aqueous methanol. Following centrifugation at 10,000 g for 10 min, the extracts were absorbed (CNWBOND Carbon-GCB SPE Cartridge, 250 mg, 3 ml; ANPEL, Shanghai, China) and filtered (SCAA-104, 0.22 μm pore size; ANPEL, Shanghai, China) before LC–MS analysis. The extract samples were analyzed using an LC–ESI–MS/MS system (HPLC, Shim-pack UFLC SHIMADZU CBM30A system; MS, Applied Biosystems 6500 Q TRAP). The analytical conditions were as follows: HPLC: column, Waters ACQUITY UPLC HSS T3 C18 (1.8 µm, 2.1 mm*100 mm); solvent system, water (0.04% acetic acid): acetonitrile (0.04% acetic acid); gradient program,95:5 V/V at 0 min, 5:95 V/V at 11.0 min, 5:95 V/V at 12.0 min, 95:5 V/V at 12.1 min, 95:5 V/V at 15.0 min; flow rate, 0.40 ml/min; temperature, 40 °C; injection volume, 2 μl. The effluent was alternatively connected to an ESI-triple quadrupole-linear ion trap (Q TRAP)-MS.

### ESI-Q TRAP-MS/MS

LIT and triple quadrupole (QQQ) scans were acquired on a triple quadrupole-linear ion trap mass spectrometer (Q TRAP), API 6500 Q TRAP LC/MS/MS System, equipped with an ESI Turbo Ion-Spray interface, operating in a positive ion mode and controlled by Analyst 1.6 software (AB Sciex). The ESI source operation parameters were as follows: ion source, turbo spray; source temperature 500 °C; ion spray voltage (IS) 5500 V; ion source gas I (GSI), gas II(GSII), and curtain gas (CUR) were set at 55, 60, and 25.0 psi, respectively; the collision gas was high. Instrument tuning and mass calibration were performed with 10 and 100 μmol/L polypropylene glycol solutions in QQQ and LIT modes, respectively. QQQ scans were acquired as MRM experiments with collision gas (nitrogen) set to 5 psi. DP and CE for individual MRM transitions was performed with further DP and CE optimization. A specific set of MRM transitions was monitored for each period according to the metabolites eluted within this period.

### RNA isolation and cDNA library construction for RNA sequencing

Total RNA was extracted from HJ seeds using the Pure RNA Isolation Kit (TIANGEN, China) following the manufacturer’s protocol. For the germinated group took 10 seeds were mixed and ground to extract RNA, and for the un-germinated group, 10 seeds were mixed and ground to extract RNA. RNA integrity was evaluated using an Agilent 2100 Bioanalyzer (Agilent Technologies, Santa Clara, CA, USA). The samples an RNA integrity number (RIN) ≥ 7 were subjected to the subsequent analysis. The total RNA from the seeds was cleaved into short fragments at the optimal temperature in a thermomixer. Then purified fragment mRNA was used to synthesize first strand cDNA and second strand cDNA. The libraries were constructed using TruSeq Stranded mRNA LTSample Prep Kit (Illumina, San Diego, CA, USA) according to the manufacturer’s instructions. Then 6 cDNA libraries were sequenced on the Illumina sequencing platform (HiSeq 2500 or Illumina HiSeq X Ten), and 125 bp/150 bp paired-end reads were generated. Raw data (raw reads) were processed using Trimmomatic. The reads containing the low-quality reads were removed too obtain the clean reads. After removing adaptor and low-quality sequences, the clean reads were assembled into expressed sequence tag clusters (contigs) and de novo assembled into transcripts by using Trinity in the paired-end method. According to the similarity and length of the sequence, the longest transcript was selected as a single gene for subsequent analysis.

### Functional annotation of differentially expressed unigenes (DEGs)

The function of the unigenes was annotated by alignment of the unigenes with the NCBI non-redundant (NR), SwissProt, and Clusters of orthologous groups for KOG databases using Blastx with a threshold E-value of 10^−5^. The proteins with the highest hits to the unigenes were used to assign functional annotations. Based on the SwissProt annotation, GO classification was performed by mapping the relation between SwissProt and GO terms. The unigenes were mapped to the Kyoto Encyclopedia of Genes and Genomes (KEGG) database to annotate their potential metabolic pathways^23,24,25^.

FPKM and read counts value of each unigene were calculated using bowtie2 and eXpress. DEGs were identified using the DESeq functions estimate SizeFactors and nbinom test. FDR adjusted p-value < 0.05 and fold change > 2 or fold change < 0.5 were set as the thresholds for significantly differential expression. Hierarchical cluster analysis of DEGs was performed to explore transcript expression patterns. GO enrichment and KEGG pathway enrichment analyses of DEGs were respectively performed using R based on the hypergeometric distribution.

### Determination of alpha-amylase activity

We obtained the radicle and CK of HJ seeds during the germination period for the determination of α-amylase activity, added 0.1 g of each sample and added 1 mL of distilled water. Next, the homogenized solution was poured into a centrifuge tube, incubated at room temperature for 20 min and subsequently shaken to complete the extraction; next, the samples were placed in a centrifuge (25 °C) and centrifuged (8000 rpm) for 10 min, and the supernatant was aspirated after centrifugation. After that step, distilled water was added to a volume of 10 mL and the solution was mixed well to obtain an amylase stock solution. The α-amylase activity was detected and calculated using an α-amylase detection kit (Suzhou Grace Biotechnology Co., Ltd., China) according to the manufacturer's protocol.

### qRT-PCR analysis

Eighteen DEGs were selected for qRT-PCR analysis, and glyceraldehyde-3-phosphate dehydrogenase (GAPDH) was used as the internal reference gene. The primers were designed based on the sequence of genes using Primer 5.0 (Premier 5.0 is a professional primer design software launched by Canadian Premier Company, with PCR or sequencing primers and hybridization probe design functions.) and are listed in Table S1. The RNA extracted from HJs was used to synthesize first-strand cDNA with HiScriptII Q RT SuperMix (Vazyme, R223-1) following the manufacturer’s instructions. qRT-PCR was performed with a SYBR Green PCR kit (Qiagen, 204,054) according to the manufacturer’s instructions. The experimental conditions were set as follows: 45 cycles at 95 °C for 20 s, 55 °C for 20 s, and 72 °C for 20 s. The mRNA expression level of the genes was calculated with the 2^−ΔΔCt^ method. Each plant sample was repeated 3 times (each replicate contained three technical replicates). The correlation between the RNA-Seq and qRT-PCR results was analyzed using the R package version 3.1.3 (http://cran.r-project.org/). The normalized values of relative expression and FPKM values were calculated using log_2_ (fold change) measurements.

### Statistical analysis of data

Data from three biological repeats were analyzed using GraphPad Prism 5, TBtools^45^, Excel 2013 and SPSS 20.0 and rendered as the means ± SD. One-way ANOVA followed by Tukey’s significant difference test at *p* < 0.05 was utilized. All data had 3 biological repeats. Differentially expressed genes were defined as genes with false discovery rate (FDR) < 0.05 and fold change > twofold. An adjusted *p* value < 0.05 was considered significant when identifying enriched GO terms, and an adjusted *p* value < 0.05 was considered indicative of significantly enriched KEGG pathways. The author confirms that all methods were performed in accordance with the relevant guidelines and regulations by the “IUCN Policy Statement on Research Involving Species at Risk of Extinction” and the “Convention on the Trade in Endangered Species of Wild Fauna and Flora”.

## Supplementary Information

Supplementary Information 1.

Supplementary Information 2.

Supplementary Information 3.
