# Supplementary material for: Transcriptome and metabolome analyses revealing the potential mechanism of seed germination in Polygonatum cyrtonema
Source: Sci Rep. 2021 Jun 9;11:12161. doi: 10.1038/s41598-021-91598-1 (PMC8190097; doi:10.1038/s41598-021-91598-1)
Supplement: Supplementary file 3 — Supplementary Information 3. [file 41598_2021_91598_MOESM3_ESM.docx]

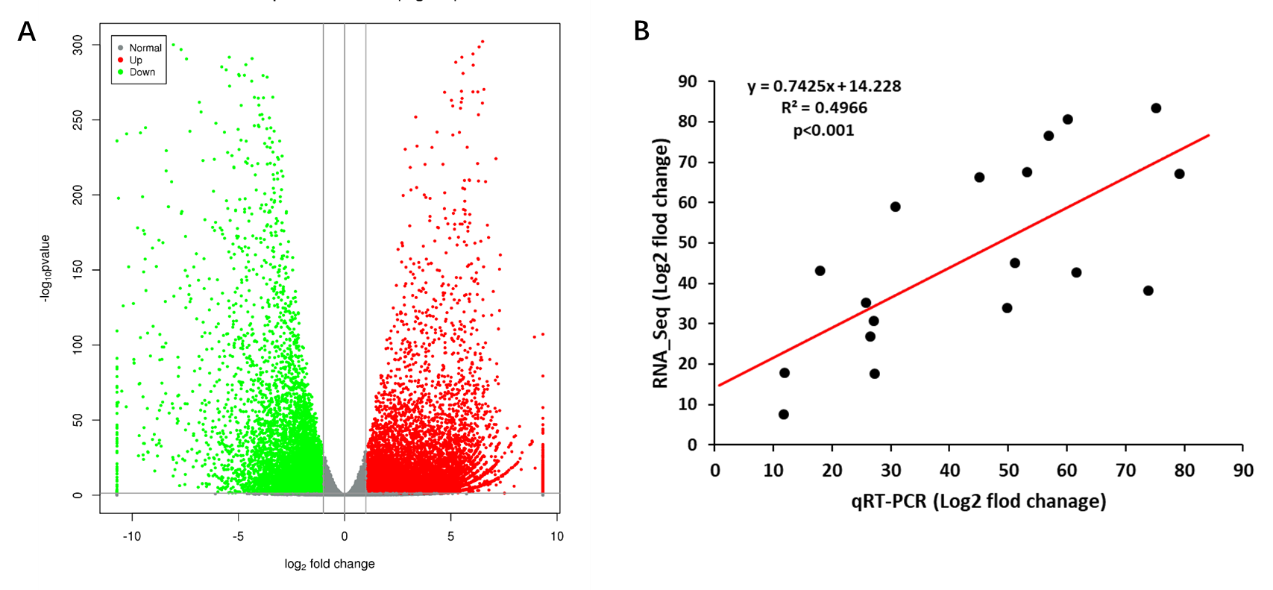


Figure S1. Volcano plots of differentially expressed unigenes (DEGs) between germinated and control seeds in *Polygonatum cyrtonema* Hua (A); and correlation between qRT-PCR and RNA_seq for the 18 genes (B). Each point represents a fold change value of gene expression level in *Polygonatum cyrtonema* Hua.


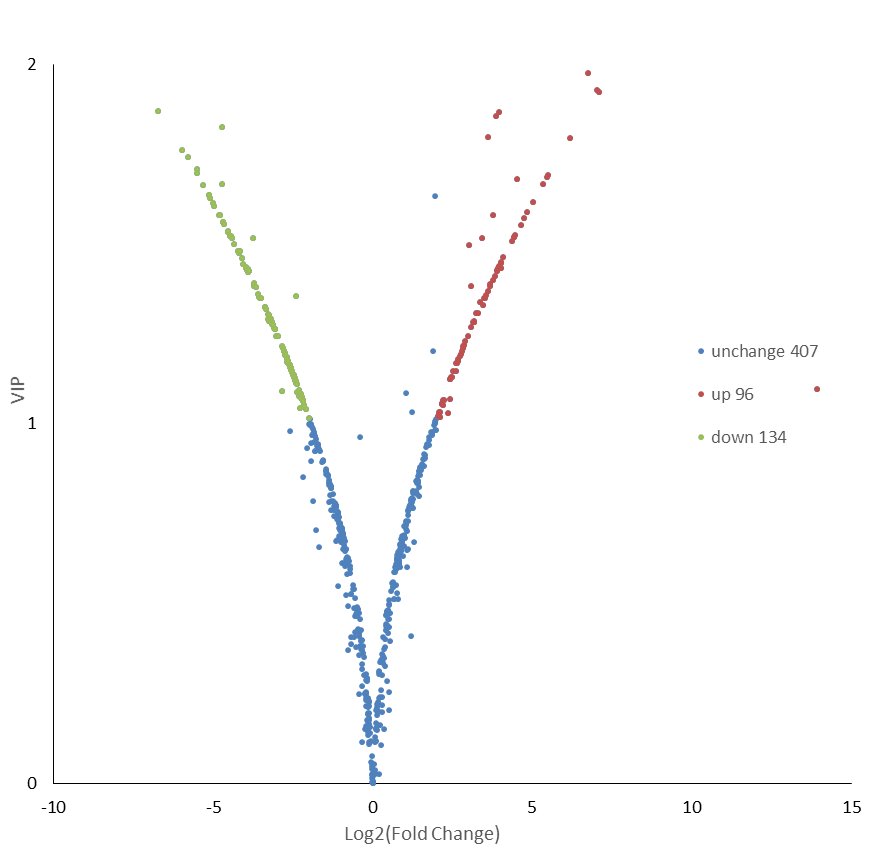


Figure S2. Volcano plots of differentially metabolites between germinated and control seeds in *Polygonatum cyrtonema* Hua.


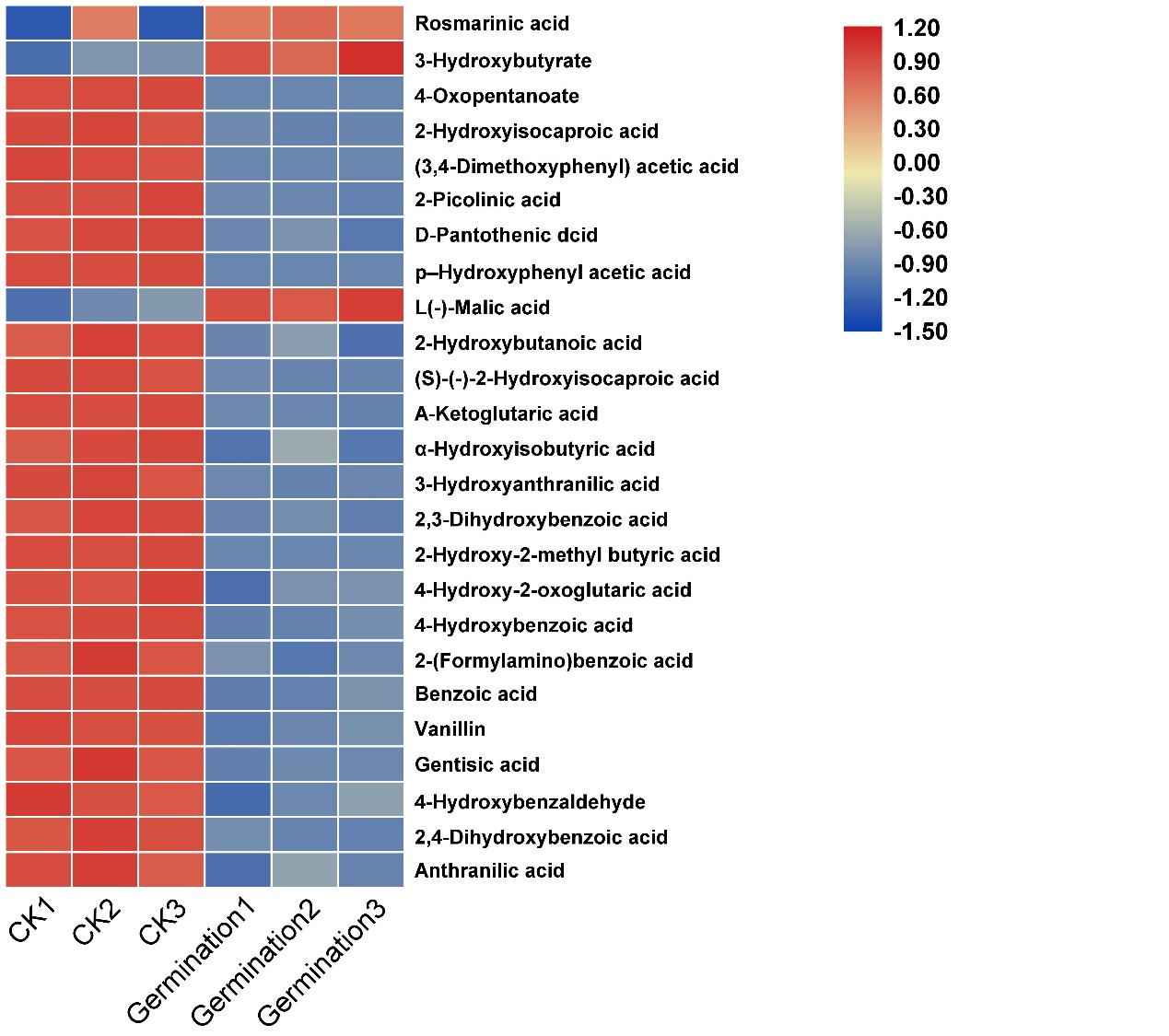


Figure S3. The differentially metabolites of organic acid between germinated and control seeds in *Polygonatum cyrtonema* Hua.
